# Dissection of 4L lymph node for left-sided non-small cell lung cancer: a meta-analysis

**DOI:** 10.3389/fonc.2025.1583508

**Published:** 2025-06-09

**Authors:** Chenxi Li, Zhuozheng Hu, Jiajun Wu, Weijun Zhou, Wenxiong Zhang, Chao Song

**Affiliations:** ^1^ Department of Thoracic Surgery, The Second Affiliated Hospital, Jiangxi Medical College, Nanchang University, Nanchang, China; ^2^ Jiangxi Medical College, Nanchang University, Nanchang, China

**Keywords:** dissection, left, 4L lymph node, non-small lung cancer, meta-analysis

## Abstract

**Background:**

The therapeutic efficacy of left lower paratracheal (4L) lymph node dissection in the management of left-sided non-small cell lung cancer (NSCLC) remains an unresolved clinical question. Therefore, we conducted a meta-analysis to compare the survival of patients with left-sided NSCLC who underwent 4L lymph node dissection (LND+) and those who did not (LND−).

**Methods:**

Seven databases were searched for relevant studies comparing patients with left-sided NSCLC who underwent 4L lymph node dissection and those who did not. The primary endpoints were survival indicators, including overall survival (OS) and disease-free survival (DFS). Secondary endpoints included hospitalization and follow-up outcomes.

**Results:**

After thoroughly screening 431 studies, six studies encompassing 4,253 patients were included in the final analysis. The LND+ group showed better OS (hazard ratio [HR]: 0.65 [0.52, 0.81], *p* < 0.0001) and DFS (HR: 0.82 [0.71, 0.95], *p* = 0.008). The 4L LND+ group also demonstrated higher OS rates at 1–5 years and DFS at 1 year. Postoperative complications and recurrence rates were similar between the two groups.

**Conclusions:**

Based on these results, 4L lymph node dissection should be performed for left-sided resectable NSCLC, due to its association with improved OS and DFS.

**Systematic review registration:**

https://www.crd.york.ac.uk/PROSPERO/view/CRD42024567681, identifier CRD42024567681.

## Introduction

Lung cancer continues to be a predominant contributor to cancer-related mortality globally (1–4). Among the subtypes of lung cancer, non-small cell lung cancer (NSCLC) accounts for the vast majority (approximately 85%) of all cases (5), and the 5-year survival rate across all stages is about 20% (6). So far, the standard treatment for early-stage NSCLC is tumor resection and lymph node dissection (7), including mediastinal lymph node dissection and systemic lymph node sampling (4, 8, 9). The National Comprehensive Cancer Network (NCCN) guidelines recommend that mediastinal lymph node dissection should include no fewer than three stations (10–12).

However, the clinical necessity of left lower paratracheal (4L) lymph node dissection (LND) for left-sided NSCLC remains unclear (10). A previous study by Wang et al. (7) showed that station 4L lymph node involvement is common in the left-sided NSCLC, and that 4L LND can improve the prognosis of patients compared to those who did not undergo this procedure (7). Another study conducted by Zhao et al. (13) demonstrated that performing 4L lymph node dissection provides greater benefits to disease-free survival (DFS) and overall survival (OS) in patients with left-sided NSCLC. Yang et al. (14) also confirmed that 4L LND improves survival in left-sided NSCLC. Similarly, Gryszko et al. (15) indicated that the benefits of lymphadenectomy are particularly evident at the 4L lymph node station. However, Wo et al. (16) presented a differing perspective, finding that 4L LND does not improve patient survival and may instead increase postoperative complications (16).

To further confirm whether performing 4L lymph node dissection improves survival in left-sided NSCLC, we conducted a meta-analysis comparing survival outcomes between patients who underwent 4L LND and those who did not.

## Materials and methods

Adhering to the Preferred Reporting Items for Systematic Reviews and Meta-Analyses (PRISMA) guidelines, this study was meticulously conducted (**Supplementary Table S1**; PROSPERO ID: CRD42024567681).

### Search strategy

We systematically searched the databases PubMed, Web of Science, EMBASE, Cochrane Library, ScienceDirect, and Scopus up to 15 April 2025, to analyze the survival of patients with left-sided NSCLC. The following MeSH terms were used: “left-sided”, “4L lymph node,” and “lung cancer”. References from the retrieved articles (including meta-analyses and abstracts) were also screened for additional eligible articles. Detailed search strategies are provided in **Supplementary Table S2**.

### Selection criteria

Inclusion criteria were as follows:

1. Population: patients with left-sided NSCLC who underwent tumor resection and LND.
2. Intervention and comparison: patients who underwent 4L LND compared with those who did not.
3. Outcomes: OS, DFS, 1–5-year overall survival rates (1–5-year OSR), 1–5-year disease-free survival rates (1–5-year DFSR), and adverse events (AEs).
4. Study design: high-quality cohort and retrospective studies.

Conference abstracts, articles without original data, animal experiments, and abstracts only were excluded.

### Data extraction

Two investigators independently extracted the following data: publication year, first author, country, number of participants, tumor characteristics (location, pathological stage), study design, participants characteristics (sex, age), lymph node metastasis, TNM stage, antitumor efficacy indices (OS, DFS, 1–5-year OSR, 1–5-year DFSR), and AEs. Any disagreements were resolved by a third investigator.

### Outcome assessment

We analyzed survival data (OS and DFS), as well as survival rates at 1–5 years (OSR and DFSR). In addition, subgroup analyses of OS and DFS were performed based on age, sex, and pathological TNM stage.

### Quality assessment

We used the Newcastle-Ottawa Scale (NOS) to assess the quality of cohort studies, a tool specifically designed for evaluating nonrandomized studies in meta-analyses. The scale includes three items: selection of groups, comparability of groups, and assessment of outcomes. Scores for the included studies were calculated (**Supplementary Table S3**) and categorized into three levels: low (0–3), moderate (4–6), and high (7–9) quality (17).

The Grading of Recommendations Assessment, Development, and Evaluation (GRADE) system was used to assess the quality of evidence for the results. This system evaluates five domains: imprecision, risk of bias, indirectness, inconsistency, and publication bias. The quality of evidence was classified into four levels: very low, low, moderate, and high (18) (**Supplementary Table S4**).

### Statistical analysis

Review Manager 5.3 and STATA 12.0 were used to analyze the pooled data in this meta-analysis. Hazard ratios were used to evaluate survival outcomes (OS and DFS). An HR > 1 favored the 4L LND− group. while an HR < 1 favored the 4L LND+ group. Heterogeneity was assessed using the *I* ^2^ statistic and the *χ* ^2^ test. A random-effects model was applied when significant heterogeneity was present (*I* ^2^ > 50% or *p* < 0.1); otherwise, a fixed-effects model was used. Begg’s rank correlation and Egger’s linear regression test were used to assess publication bias. *p* < 0.05 was considered statistically significant.

## Result

### Search results and study quality assessment

**Figure 1:**
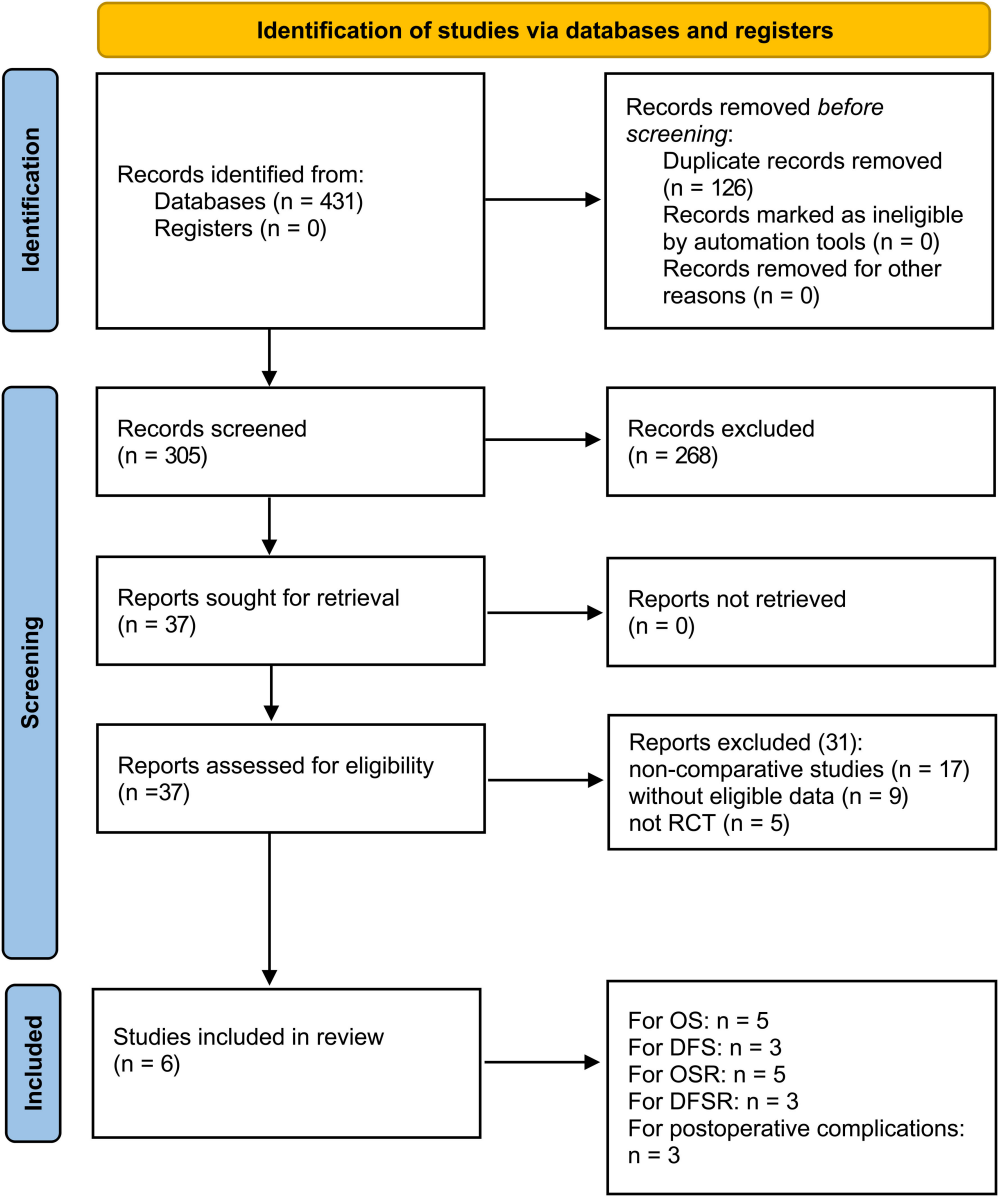
Flowchart of the study selection process.

**Figure 1** illustrates the entire process of literature screening for the meta-analysis. A total of 431 eligible studies were initially identified. After a systematic search, six studies involving 4,253 patients were included in the final analysis (1,986 patients in the 4L LND+ group and 2,267 patients in the 4L LND− group). Among these, five studies (7, 13, 14, 16, 22) were conducted in China, and one study (15) was conducted in Poland. All patients in the included studies underwent surgical resection. All of the included studies were retrospective cohort studies, and propensity score matching (PSM) was applied in each to minimize potential bias (sex, age, TNM stage, surgical procedure, tumor location, tumor size, histological type, etc.).

As for the NOS, PSM was performed in all of the included studies; thus, the groups in these studies were well-balanced. Therefore, the scores of the five included studies were all > 7, indicating high quality. The baseline characteristics of the included studies are presented in **Table 1**. The GRADE system showed that most of the studies were of high quality (**Supplementary Table S4**).

**Table 1:** Summary of baseline characteristics of the included studies.

Year	Author	Country	Treatment arms	Patients (*n*)	Age (mean, years)	Patients (*n*) after PSM	Sex (M/F)	Adjustment for confounding factors	4L LN metastasis rate	Tumor type	pTNM stage	Follow‐up time (m)	Study design
2023	Wu (22)	China	4L LND+; 4L LND−	119; 193	NA	119; 193	70/49; 17/76	Age, sex, smoking history, clinical stage, adjuvant therapy, tumor differentiation, and tumor size were well balanced between the two groups	9.2	NSCLC	I–III	77	Cohort study
2022	Wo (16)	China	4L LND+; L LND−	586; 54	50.4; 0.3	416; 16	278/138; 269/147	Sex, histologic subtype, T stage, age, smoking history, LVI status, location, APLN status, IMLN status, N1 LN status, and surgical procedure	16.6	NSCLC	I–III	77	Cohort study
2021	Gryszko (15)	Poland	4L LND+; L LND−	659; 4,710	62.4; 62.9	659; 659	489/170; 475/184	Age, sex, smoking history, histopathological recognition, stage of lung cancer, and pathological T stage	10	NSCLC	0–IIIB	60.8	Cohort study
2020	Yang (14)	China	4L LND+; 4L LND−	391; 1,538	58.8 ± 9.9; 59.3 ± 10.2	317; 17	214/103; 33/84	Sex, age, tumor location, tumor size, anatomical type, smoking history, and surgical procedure, histology, cell differentiation, adjuvant therapy, pT category, pN category, and the number of resected lymph nodes	11.8	NSCLC	T1–4N0–M0	60 (range: 1–208)	Cohort study
2019	Zhao (13)	China	4L LND+; L LND−	460; 604	58.4	460; 460	309/151; 305/155	Age, sex, smoking history, tumor location, tumor size, histologic type, pathologic N (pN) stage, and surgical approach	14.6	NSCLC	I–IIIA	40	Cohort study
2018	Wang (7)	China	4L LND+; L LND−	139; 518	NA	134; 415	94/40; 284/131	Age, sex, pathological T (pT) stage, smoking history, pathological N (pN) stage, histology, tumor location, tumor area, and pathological tumor-node-metastasis (pTNM) stage	20.9	NSCLC	I–IIIB	99 (range: 4–153)	Cohort study

*4L*, left lower paratracheal; *LND*, lymph node dissection; *LND+*, patients with lymph node dissection; *LND−*, patients without lymph node dissection; *PSM*, propensity score matching; *LVI*, lymphovascular invasion; *APLN*, aortopulmonary zone lymph nodes; *IMLN*, inferior mediastinal lymph nodes; *pTNM*, pathological tumor node metastasis; *NSCLC*, non-small cell lung cancer.

### Survival

**Figure 2:**
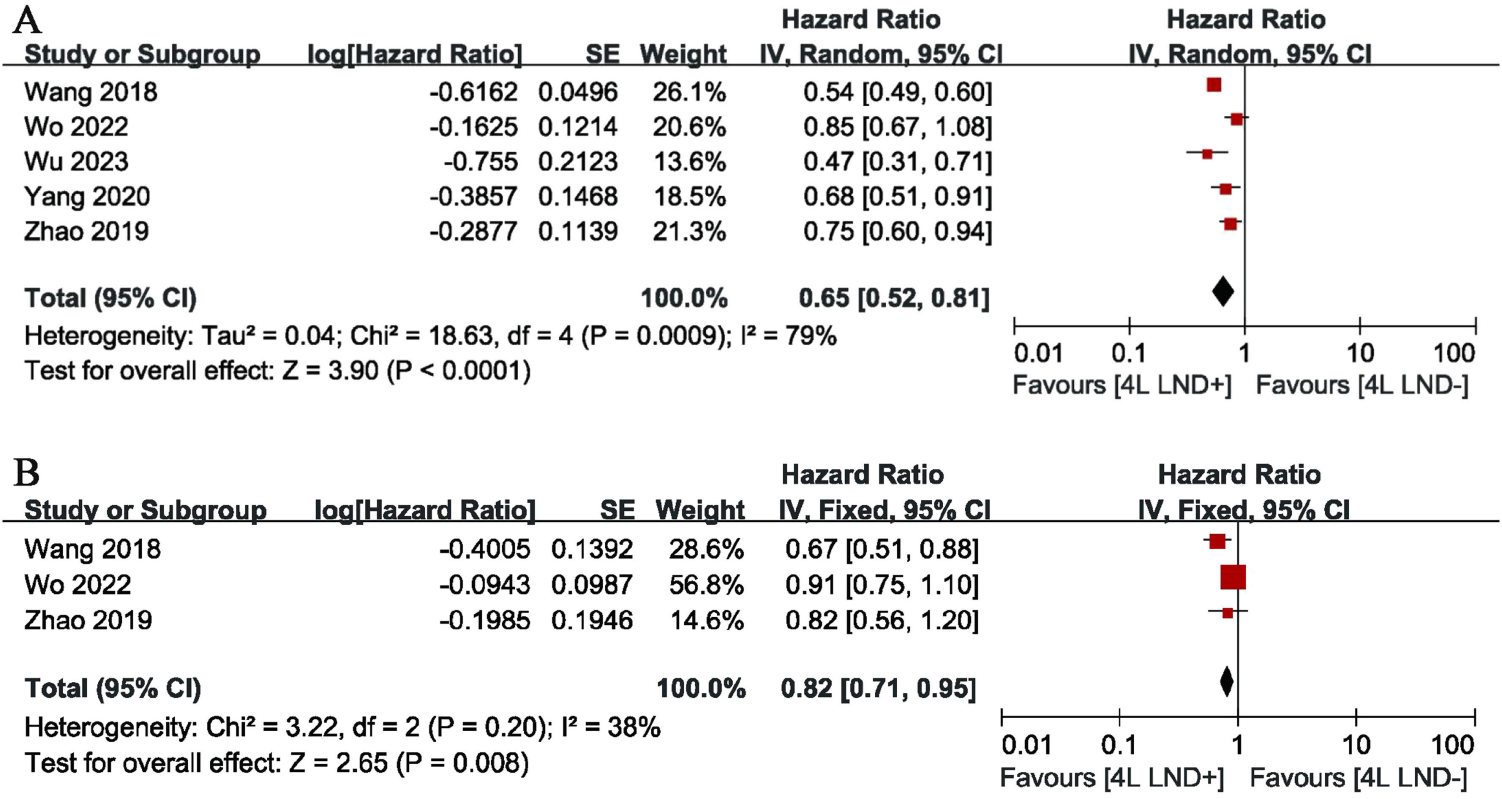
Forest plots of OS **(A)** and DFS **(B)** comparing 4L LND+ and 4L LND−.

**Figure 3:**
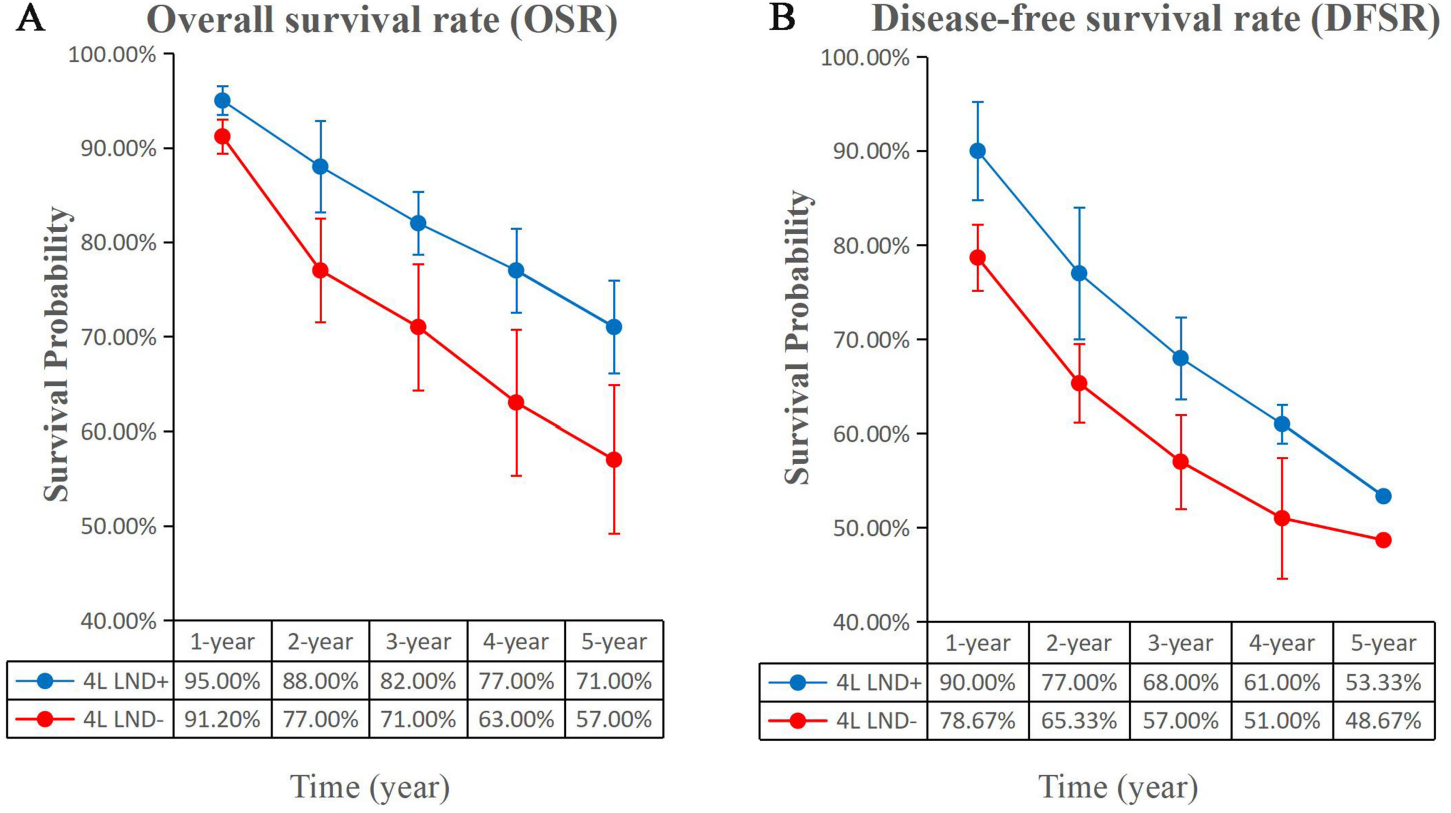
Comparisons of OSR (1–5 years, **A**) and DFSR (1–5 years, **B**) between 4L LND+ and 4L LND− groups.

**Figure 4:**
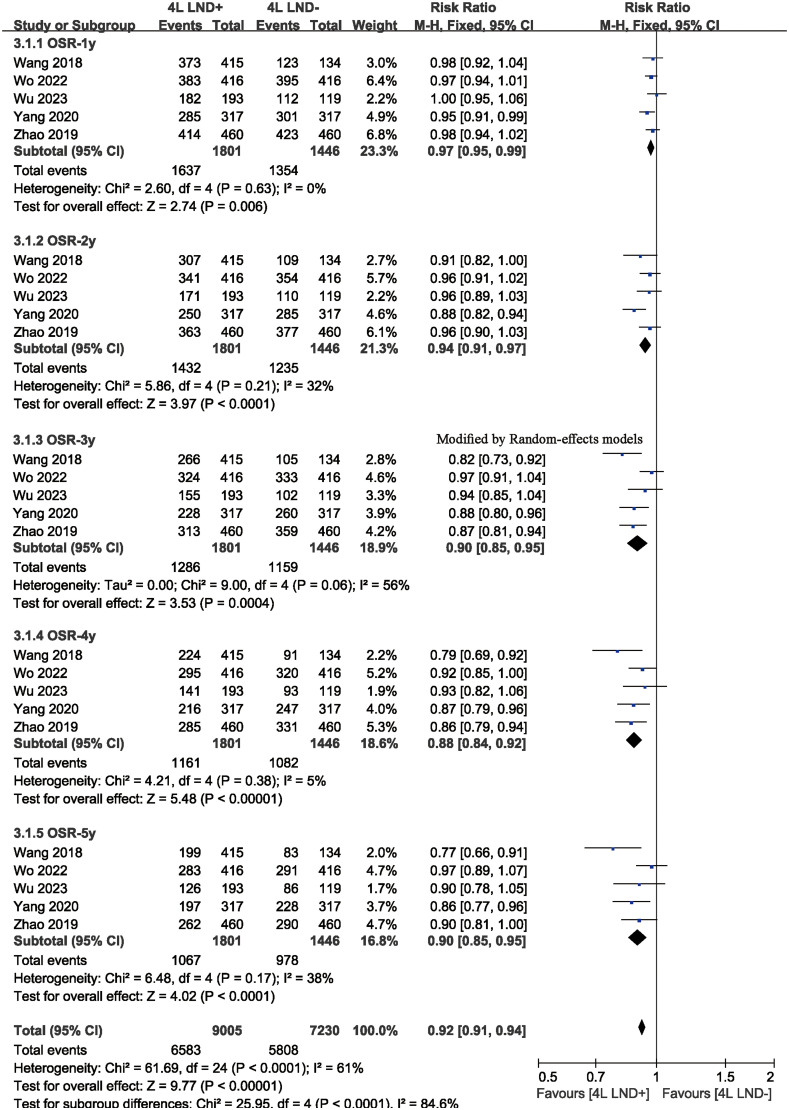
Comparisons of OSR (1–5 years) between 4L LND+ and 4L LND− groups by survival time.

**Figure 5:**
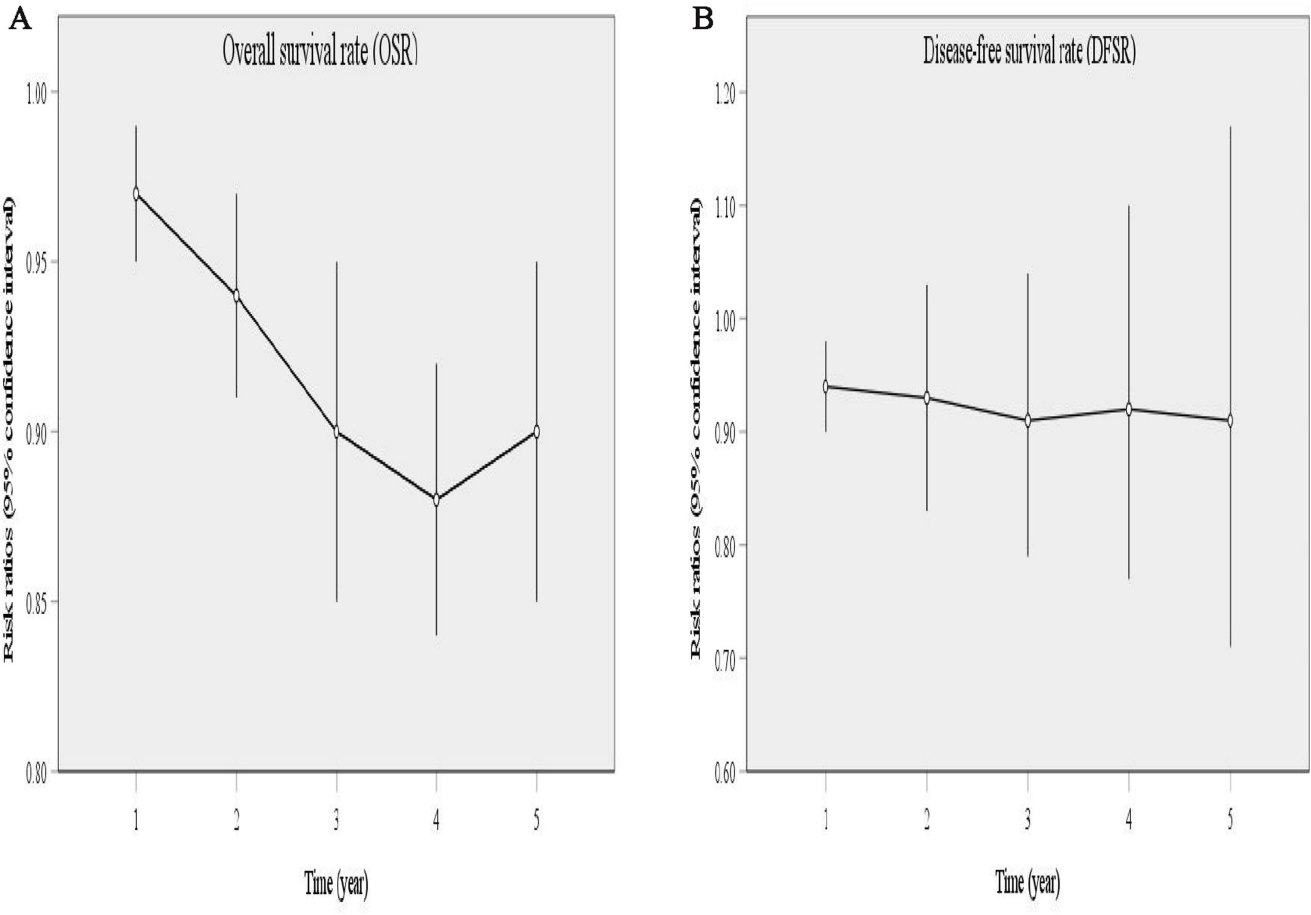
Line charts of OSR (1–5 years, **A**) and DFSR (1–5 years, **B**) comparing 4L LND+ and 4L LND− groups by survival time.

Five studies compared OS, showing high heterogeneity (*p* = 0.0009, *I* ^2^ = 79). The results indicated that, compared with the 4L LND− group, patients who underwent 4L lymph node dissection had significantly better OS (HR: 0.65 [0.52, 0.81], *p* < 0.0001) (**Figure 2A**). Subgroup analysis demonstrated that the 4L LND+ group achieved better OSR-1y (RR: 0.97 [0.95, 0.99], *p* = 0.006), OSR-2y (RR: 0.94 [0.91, 0.97], *p* < 0.00001), OSR-3y (RR: 0.90 [0.85, 0.95], *p* = 0.0004), OSR-4y (RR: 0.88 [0.84, 0.92], *p* < 0.00001), and OSR-5y (RR: 0.90 [0.85, 0.95], *p* < 0.0001) (**Figures 3A**, **4**). With the prolongation of survival time, the advantage of OSR in the 4L LND+ group became more apparent (**Figure 5A**). Subgroup analysis based on tumor location showed that in both the left upper lobe (LUL) and left lower lobe (LLL), the 4L LND+ group tended to achieve better 1–5-year OSR (**Supplementary Figures S1A, B**).

**Figure 6:**
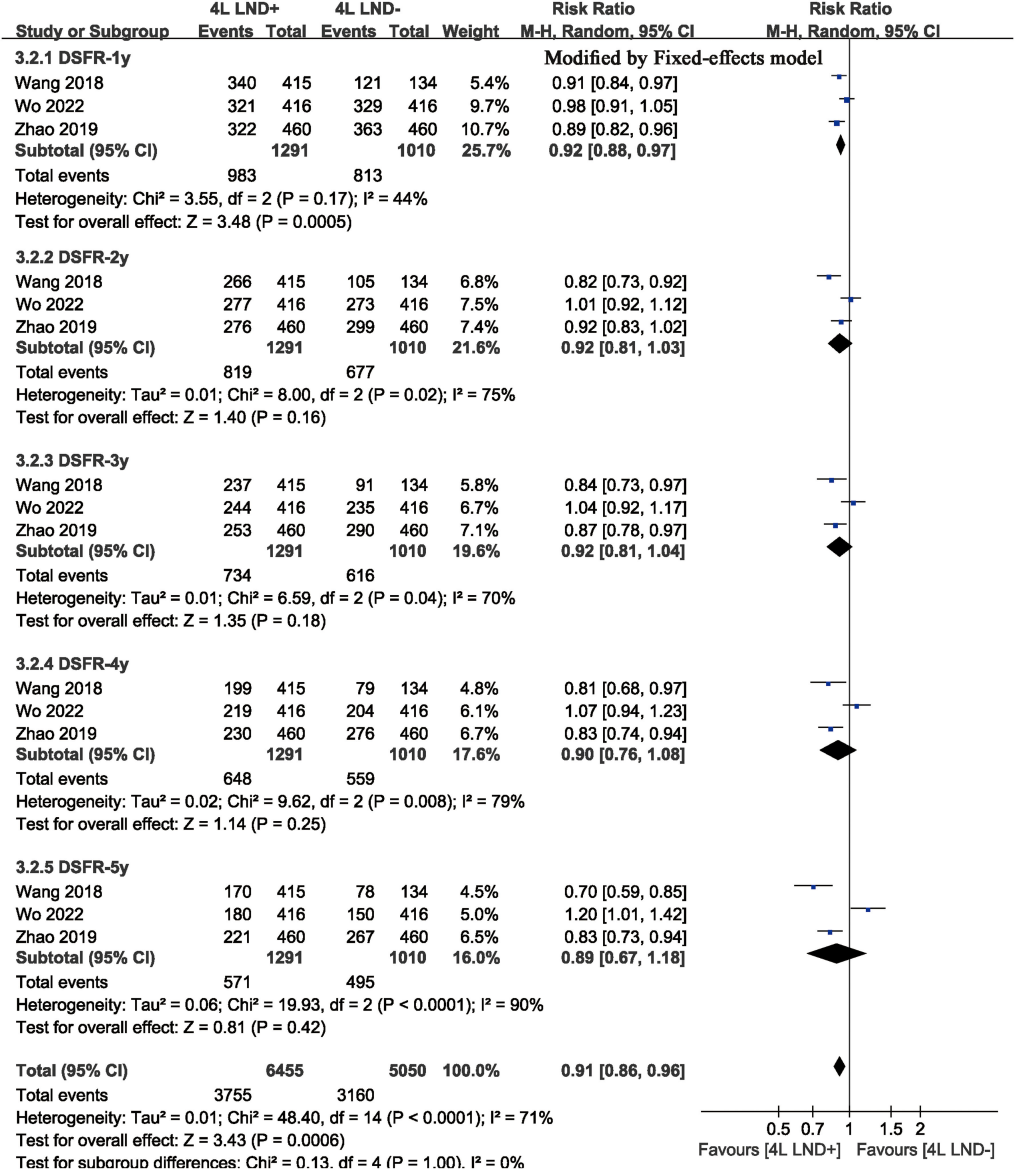
Comparisons of DFSR (1–5 years) between 4L LND+ and 4L LND− groups by survival time.

Two studies compared DFS (heterogeneity: *p* = 0.20, *I* ^2^ = 38). The results showed that the 4L LND+ group had better DFS (HR: 0.82 [0.71, 0.95], *p* = 0.008) (**Figure 2B**). Subgroup analysis indicated that the 4L LND+ group achieved better DFSR-1y (RR: 0.92 [0.88, 0.97], *p* = 0.0005), DFSR-2y (RR: 0.92 [0.81, 1.03], *p* = 0.16), DFSR-3y (RR: 0.92 [0.81, 1.04], *p* = 0.18), DFSR-4y (RR: 0.90 [0.76, 1.08], *p* = 0.25), and DFSR-5y (RR: 0.89 [0.67, 1.18], *p* = 0.42) (**Figures 3B**, **6**). As survival time increased, the advantage of DFSR in the 4L LND+ group became more apparent (**Figure 5B**).

### Subgroup analysis

We evaluated the possible factors that may affect the survival of the 4L LND+ group compared to the 4L LND− group in early-stage left-sided NSCLC. The results suggested that the 4L LND+ group had a more favorable impact on survival (**Table 2**).

**Table 2:** Subgroup analysis of survival (OS and DFS) comparing 4L LND+ vs. 4L LND− in patients undergoing lobectomy.

Subgroups	No. of studies	Overall survival		No. of studies	Disease-free survival	
		HR (95% CI)	*p*-value		HR (95% CI)	*p*-value
**Total**	5	0.65 [0.52, 0.81]	< 0.0001	3	0.92 [0.66, 1.29]	0.64
Published year						
Earlier than 2020	2	0.57 [0.52, 0.62]	< 0.00001	2	1.01 [0.87, 1.19]	0.86
2020–2022	3	0.72 [0.60, 0.85]	< 0.0001	1	0.91 [0.75, 1.10]	0.34
Nation						
China	5	0.60 [0.55, 0.65]	< 0.00001	3	0.97 [0.86, 1.10]	0.64
Poland	–	–	–	–	–	–
Follow-up time						
£60 month	2	0.72 [0.61, 0.86]	0.0003	1	1.25 [1.03, 1.52]	0.02
> 60 month	3	0.57 [0.52, 0.62]	< 0.00001	2	0.82 [0.70, 0.96]	0.01

*OS*, overall survival; *DFS*, disease-free survival; *HR*, hazard ratio; *CI*, confidence interval.

When the HR > 1, the results supported the 4L LND− group.

### Hospitalization and follow-up indicators

Postoperative hospital stay (mean difference [MD]: 0.32 [0.14, 0.50] days, *p* = 0.0005, **Supplementary Figure S2**) was similar between the two groups. Postoperative complications are shown in **Table 3**. The results indicated that the incidence of complications was also similar between the two groups (RR: 1.45 [1.01, 2.08], *p* = 0.04) (**Supplementary Figure S3**). Similarly, overall recurrences (RR: 0.49 [0.11, 2.24], *p* = 0.36), local LN recurrences (RR: 0.73 [0.47, 1.15], *p* = 0.17), and supraclavicular or cervical LN recurrences (RR: 0.79 [0.36, 1.71], *p* = 0.54) were not significantly different between the two groups (**Supplementary Figure S4**). Subgroup analysis showed that patients in the 4L LND+ group were more likely to experience locoregional recurrence, whereas patients in the 4L LND− group were more likely to experience distant metastasis or locoregional recurrence and distant metastasis (**Supplementary Figure S5**).

**Table 3:** Total adverse events according to the combination of two groups.

Adverse effects	Studies involved	4L LND+		4L LND−		Total incidence	Risk ratio	95% CI	*p*-value
		Event/Total	%	Event/Total	%				
Total	2	60/535	11.21%	51/609	8.37%	9.70%	1.45	1.01–2.08	0.04
Chylothorax	3	13/995	1.31%	11/1,069	1.03%	1.16%	1.28	0.58–2.84	0.54
Pneumonia	3	35/995	3.52%	28/1,069	2.62%	3.05%	1.29	0.79–2.10	0.31
Hemorrhage	2	8/535	1.50%	2/609	0.33%	0.87%	4.67	0.93–23.46	0.06
Air leak > 7 days	1	10/416	2.40%	7/416	1.68%	2.04%	1.43	0.55–3.72	0.46
Chest tube drain > 7 days	2	65/876	7.42%	49/876	5.59%	6.51%	0.98	0.31–3.08	0.97
Heart failure	1	2/416	0.48%	2/416	0.48%	0.48%	1	0.14–7.07	1
Recurrent nerve injury	1	3/416	0.72%	1/416	0.24%	0.48%	3	0.31–28.72	0.34
Hoarseness	1	5/460	1.09%	4/460	0.87%	0.98%	1.25	0.34–4.63	0.74
Bronchopleural fistula	2	5/579	0.86%	5/653	0.77%	0.81%	1.1	0.32–3.76	0.88
Deep venous thrombosis	1	1/460	0.22%	3/460	0.65%	0.43%	0.33	0.03–3.19	0.34
Pulmonary embolism	1	1/460	0.22%	1/460	0.22%	0.22%	1	0.06–15.94	1
Arrhythmia	1	2/119	1.68%	3/193	1.55%	1.60%	1.08	0.18–6.38	0.93
Respiratory failure	1	2/119	1.68%	2/193	1.04%	1.28%	1.62	0.23–11.36	0.63
Pneumothorax	1	7/119	5.88%	8/193	4.15%	4.81%	1.42	0.53–3.81	0.49
Incision infection	1	1/119	0.84%	0/193	0	0.32%	4.85	0.20–118.09	0.33
Hydrothorax	1	1/119	0.84%	4/193	2.07%	1.60%	0.41	0.05–3.58	0.42
Others	1	4/119	3.36%	2/193	1.04%	1.92%	3.24	0.60–17.44	0.17

*4L*, center lower paratracheal; *LND*, lymph node dissection; *LND+*, patients with lymph node dissection; *LND−*, patients without lymph node dissection; *CI*, confidence interval.

### Occurrence

We analyzed the incidence and distribution of mediastinal lymph node metastasis according to tumor location. The results showed that station L4 had a similar occurrence rate between the LUL and LLL. Metastasis in stations 5 and 6 was more common in LUL, whereas stations 7 and 8 were more frequently involved in LLL (**Table 4**).

**Table 4:** Comparison of occurrence and distribution of mediastinal lymph node metastasis by LN station between LLL and LUL in the entire cohort.

Station	LN metastatic rate (%; involved/resected)		
	Total	LUL	LLL
L4	11.2 (57/510)	11.5 (39/338)	13.6 (18/132)
5	15.9 (297/1,870)	20.6 (234/1,136)	8.6 (63/734)
6	13.4 (122/911)	18.7 (99/530)	6 (23/381)
7	11.5 (199/1,730)	4.4 (44/995)	21 (155/735)
8	5.2 (12/233)	1.8 (2/113)	11.2 (10/89)
9	6.1 (88/1,433)	1.7 (14/814)	11.9 (74/619)
10	10.4 (24/230)	7.6 (11/145)	15.3 (13/85)
11	13.9 (35/251)	7.7 (11/143)	22 (24/109)
12	11.6 (25/215)	6.6 (8/122)	18.3 (17/93)
13	11 (18/164)	7.4 (7/95)	15.9 (11/69)

*LUL*, center upper lobe; *LLL*, center lower lobe.

### Sensitivity analysis

We performed sensitivity analyses for OS and DFS (**Supplementary Figure S6**). To assess the sensitivity and reliability of the results, we evaluated the impact of each study on the overall outcomes, which indicated that the OS and DFS findings were reliable and stable.

### Publication bias

No publication bias was detected in OS and DFS (**Supplementary Figure S7**).

## Discussion

Lung cancer remains a leading cause of cancer-related death worldwide (19). Among the various types, NSCLC accounts for a large proportion, representing approximately 80%–85% of all lung cancer cases (20). Currently, the standard treatment for resectable NSCLC is surgical resection combined with lymph node dissection (8, 9). However, for left-sided NSCLC, the necessity of dissecting the 4L lymph nodes remains clinically uncertain (10, 21, 22), with the exception of the European Society of Thoracic Surgeons guidelines, which recommend 4L LND for left-sided NSCLC (11). Therefore, we conducted a systematic review and meta-analysis to evaluate whether performing 4L lymph node dissection improves survival time and prognosis in patients with resectable left-sided NSCLC based on previous related studies. The results demonstrated that, compared to patients who did not undergo 4L lymph node dissection, those in the 4L LND+ group had significantly better OS and DFS. Additionally, the 1–5-year survival rates for both OS and DFS were higher in the 4L LND+ group. Postoperative hospital stay, complications, and overall recurrence rates were similar between the two groups.

Better survival was the most significant advantage observed in the 4L LND+ group compared to the 4L LND− group. The primary endpoints of this meta-analysis were OS and DFS. Patients who underwent 4L LND+ dissection demonstrated superior outcomes compared to those who did not. Five studies assessed OS, showing a clear increase in survival for the 4L lymph node dissection group. Additionally, two studies compared DFS between the groups, with results indicating a tendency toward improved DFS in the 4L LND+ group. We also assessed the 1–5-year OS rate and DFS rates. The 4L LND+ group showed higher OS rates across all 5 years. Similarly, the 1–5-year DFS rates tended to be better in the 4L LND+ group. However, one study by Wang et al. reported contrary findings, showing that patients who did not undergo 4L lymph node dissection achieve better 1–5-year OS rates (7). Regarding the 1–5-year DFS rates, two studies made comparisons, and the results indicated that patients in the 4L LND+ group tended to have DFS rates over the 5-year period.

Both the postoperative hospital stay and the incidence of postoperative complications were similar between the two groups. Several factors may account for this finding. First, the similarity in hospital stay is likely due to the fact that 4L lymph node dissection does not significantly increase surgical trauma or impede postoperative recovery. The dissection itself is minimally disruptive to critical intrathoracic structures (e.g., aorta, thoracic ducts, etc.) when performed using standardized surgical techniques. Additionally, baseline characteristics (e.g., pulmonary function, surgical approach, anesthetic management, etc.) were likely comparable between the groups. Furthermore, standardized postoperative nursing care and rehabilitation protocols may have contributed to consistent hospitalization durations, regardless of whether 4L lymph node dissection was performed. The similarity in the incidence of postoperative complications is likely due to the fact that 4L lymph node dissection did not significantly increase the risk of intraoperative injuries (e.g., to the thoracic duct, aorta, or recurrent laryngeal nerve) when performed using standardized surgical techniques. Additionally, the anatomical structure of the left 4L region is relatively stable, and the dissection technique is well established. Moreover, preoperative evaluation, surgical scope, and perioperative management were likely comparable between the two groups, which may have contributed to the similar incidence of common complications such as infections, coeliac chest, or hemorrhage, regardless of whether 4L lymph node dissection was performed.

The discrepancies between our findings and previous studies may stem from differences in research design, sample characteristics, and methodological approaches. For instance, while Deng et al. focused on the Chinese population, our study enrolled a Polish population, which could influence outcomes due to ethic differences (23). Additionally, unlike studies that employed RCTs, our study included cohort studies, which may affect the credibility of the results. Variations in sample size (e.g., Deng et al.: *n* = 2,103 vs. our study: *n* = 4,253) may further account for the divergent results. These comparisons underscore the importance of contextualizing findings within study-specific parameters.

However, this study has several limitations. First, only five studies were included in the meta-analysis, which may affect its reliability and feasibility, even though all included studies were of high quality. Expanding the analysis to incorporate ongoing or recently completed RCTs or retrospective studies could offer a more comprehensive and up-to-date evaluation. Second, all studies included were published in English, which may introduce language bias. Future meta-analyses should consider incorporating studies published in multiple languages, potentially with professional translation support, to reduce selection bias. Third, since individual patient data could not be obtained, heterogeneity may exist among the included studies. Future research should aim to conduct individual patient data meta-analyses, which would enable a more personalized and precise assessment of treatment efficacy and safety. Fourth, the difference in OS between the two groups was not statistically significant, which may affect the overall conclusion. Additional studies are needed to enhance the reliability of the findings. Lastly, only two studies analyzed the DFS and 1–5-year DFS rates, which may also limit the reliability of the study. More research is required to strengthen the need to incorporate more research to improve.

## Conclusion

In summary, 4L LND+ appears to be a suitable choice for left-sided NSCLC, offering improved survival (OS and DFS) with similar rates of hospitalization, complications, and recurrence. The survival benefits associated with 4L LND+ increased over longer follow-up periods. However, due to the limitations mentioned above, these results require confirmation through additional large-sample randomized controlled trials (RCTs).

## Data Availability

The original contributions presented in the study are included in the article/**Supplementary Material**. Further inquiries can be directed to the corresponding author.

## Supplementary material

Supplementary Figure 1 Subgroup analysis of OSR (1–5 years) in LUL and LLL associated with 4L LND+ versus 4L LND- according to survival time.

Supplementary Figure 2 Comparison of postoperative hospital stay between the 4L LND+ and 4L LND- group.

Supplementary Figure 3 Forest plot of postoperative complications.

Supplementary Figure 4 Forest plot of recurrence.

Supplementary Figure 5 subgroup analysis of overall recurrence.

Supplementary Figure 6 Sensitivity analysis of OS and DFS.

Supplementary Figure 7 Publication bias of OS and DFS.

Supplementary Table 1 PRISMA 2009 Checklist.

Supplementary Table 2 Search strategy.

Supplementary Table 3 Methodological quality assessments of the included studies.

Supplementary Table 4 GRADE Quality assessment by therapeutic strategy and study design for the outcomes of survival and postoperative complications.
