# Supplementary figures and images for: Dissection of 4L lymph node for left-sided non-small cell lung cancer: a meta-analysis
Source: Front Oncol. 2025 Jun 9;15:1583508. doi: 10.3389/fonc.2025.1583508 (PMC12183196; doi:10.3389/fonc.2025.1583508)

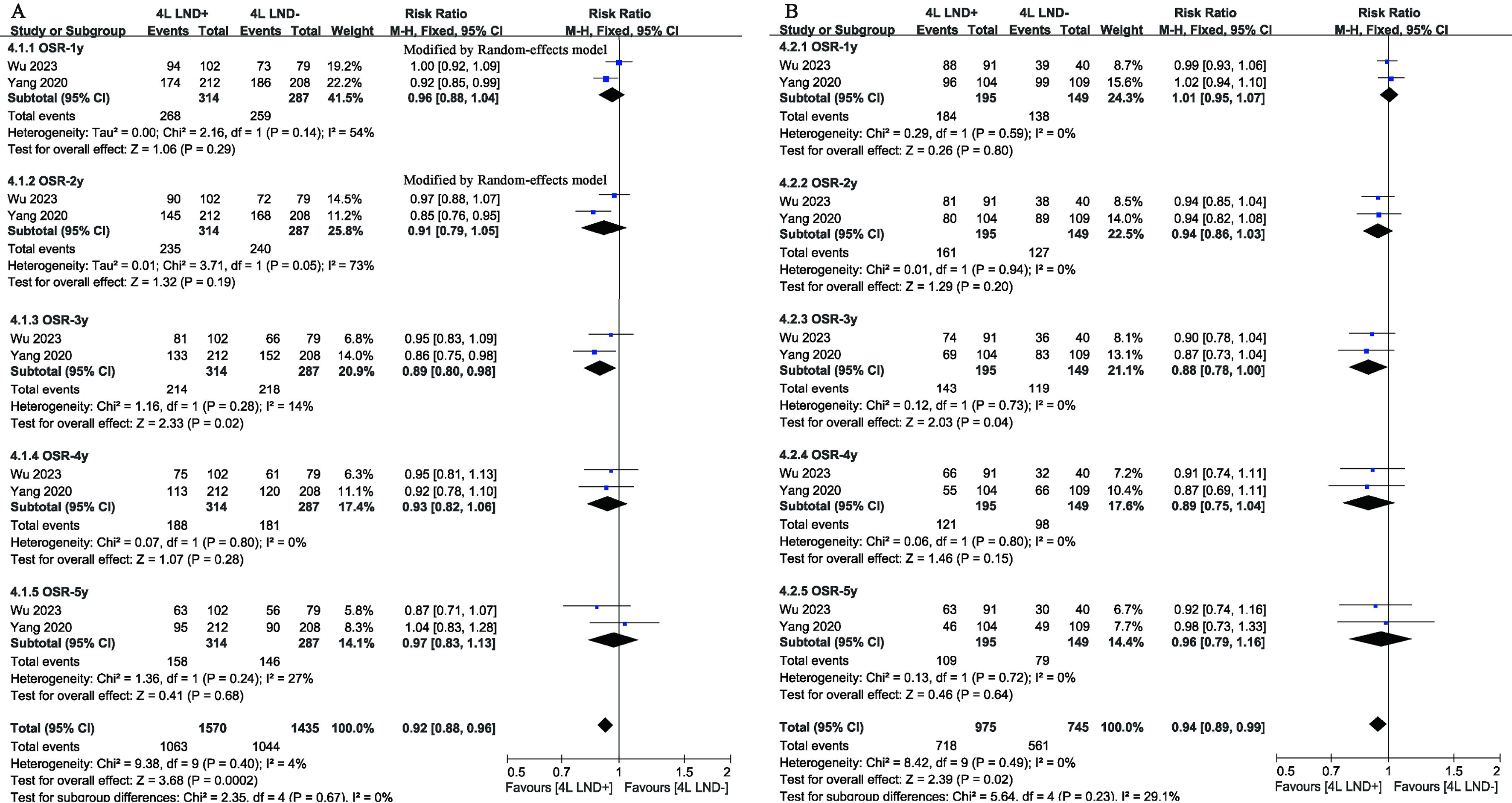

Supplement: Supplementary Figure 1 — Subgroup analysis of OSR (1–5 years) in LUL and LLL associated with 4L LND+ versus 4L LND- according to survival time. [file Image1.tif]

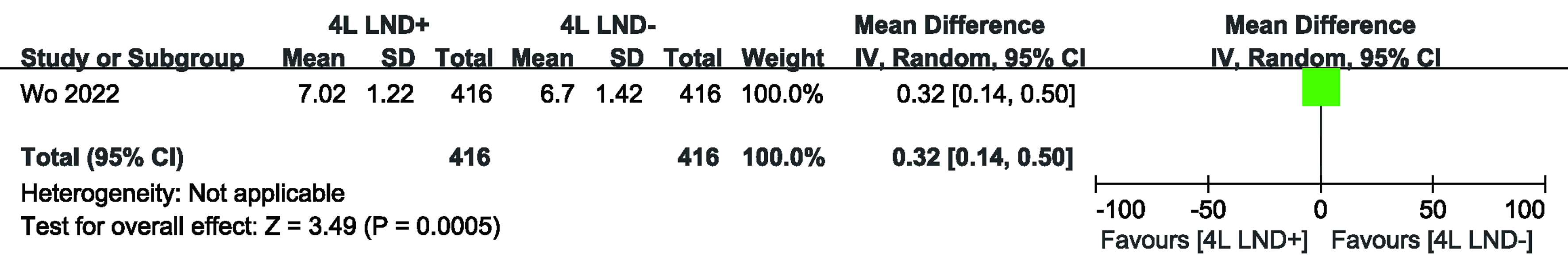

Supplement: Supplementary Figure 2 — Comparison of postoperative hospital stay between the 4L LND+ and 4L LND- group. [file Image2.tif]

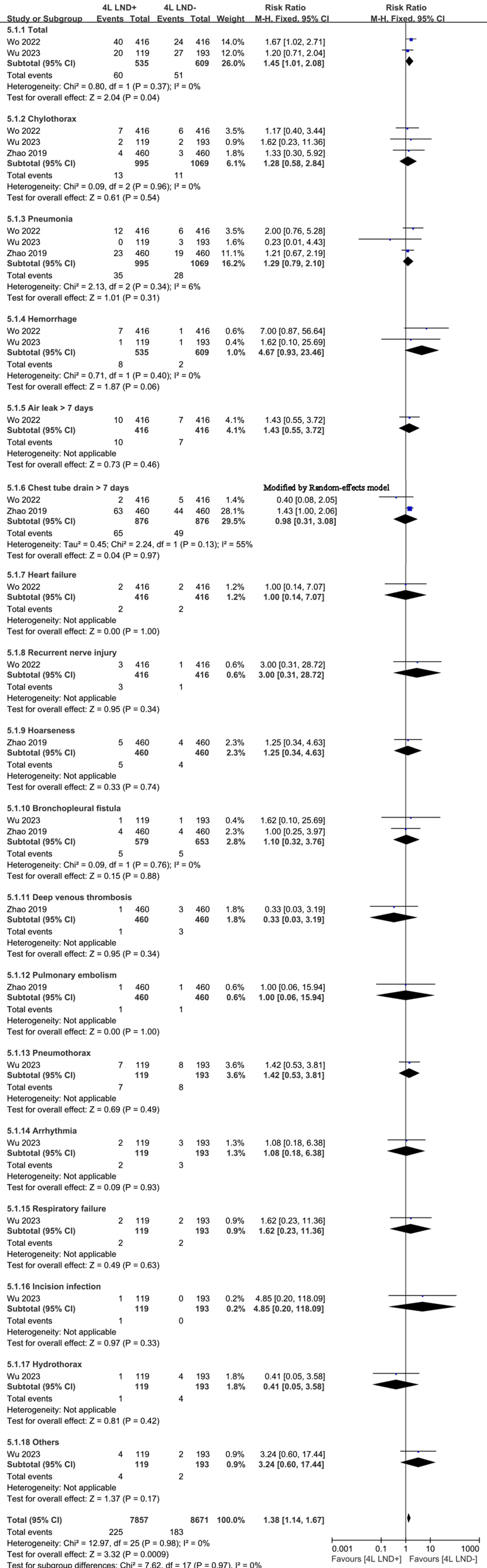

Supplement: Supplementary Figure 3 — Forest plot of postoperative complications. [file Image3.tif]

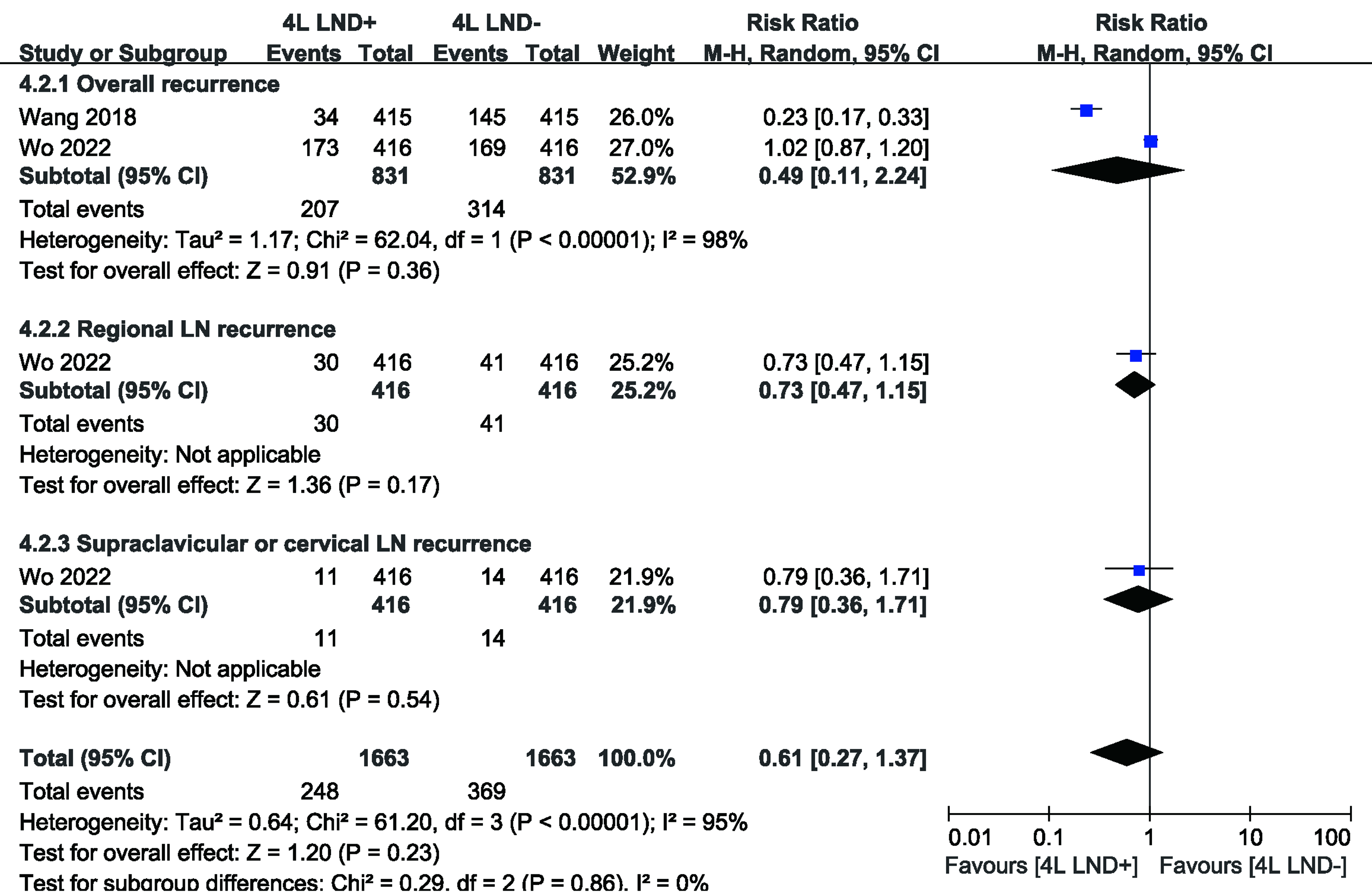

Supplement: Supplementary Figure 4 — Forest plot of recurrence. [file Image4.tif]

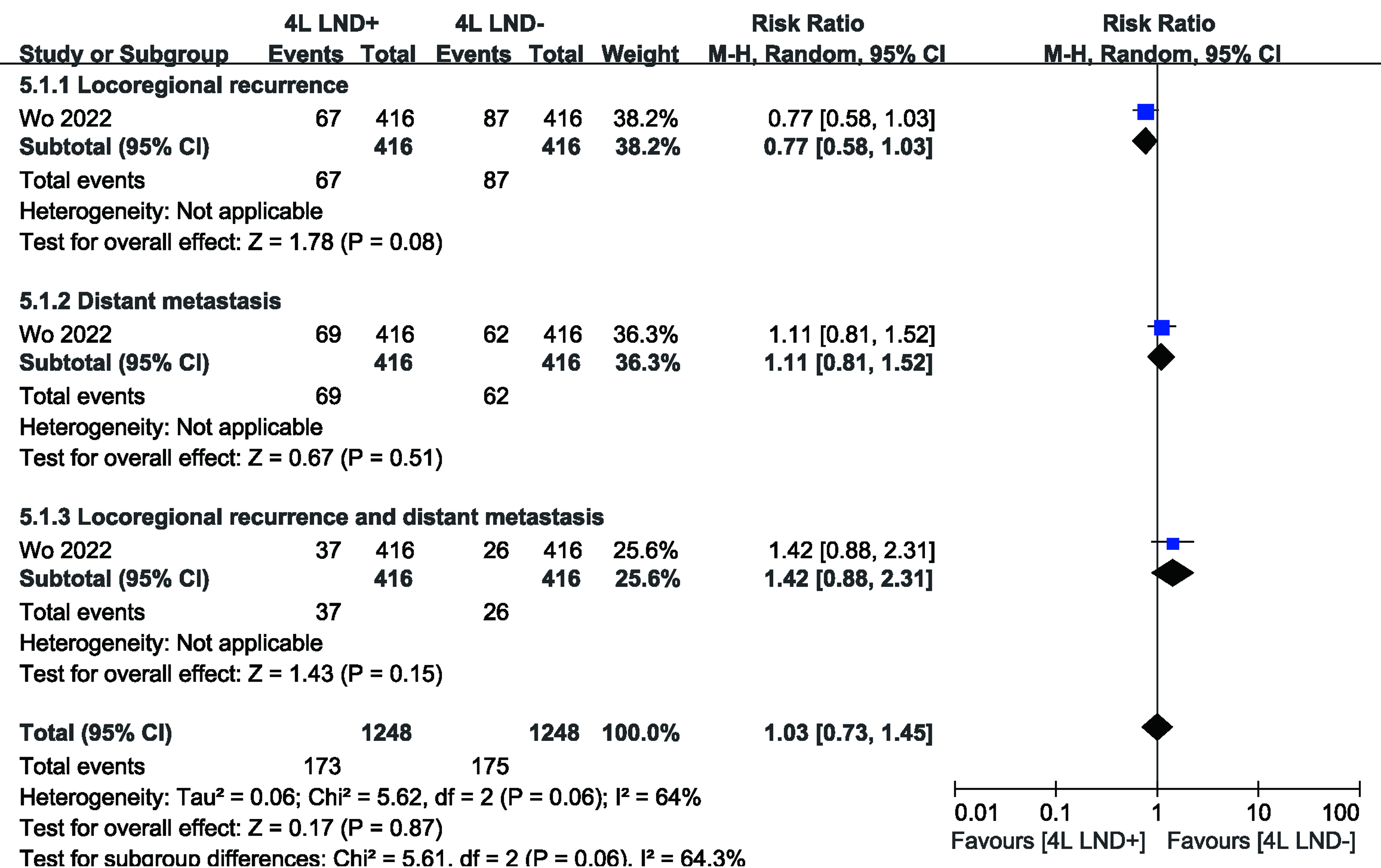

Supplement: Supplementary Figure 5 — subgroup analysis of overall recurrence. [file Image5.tif]

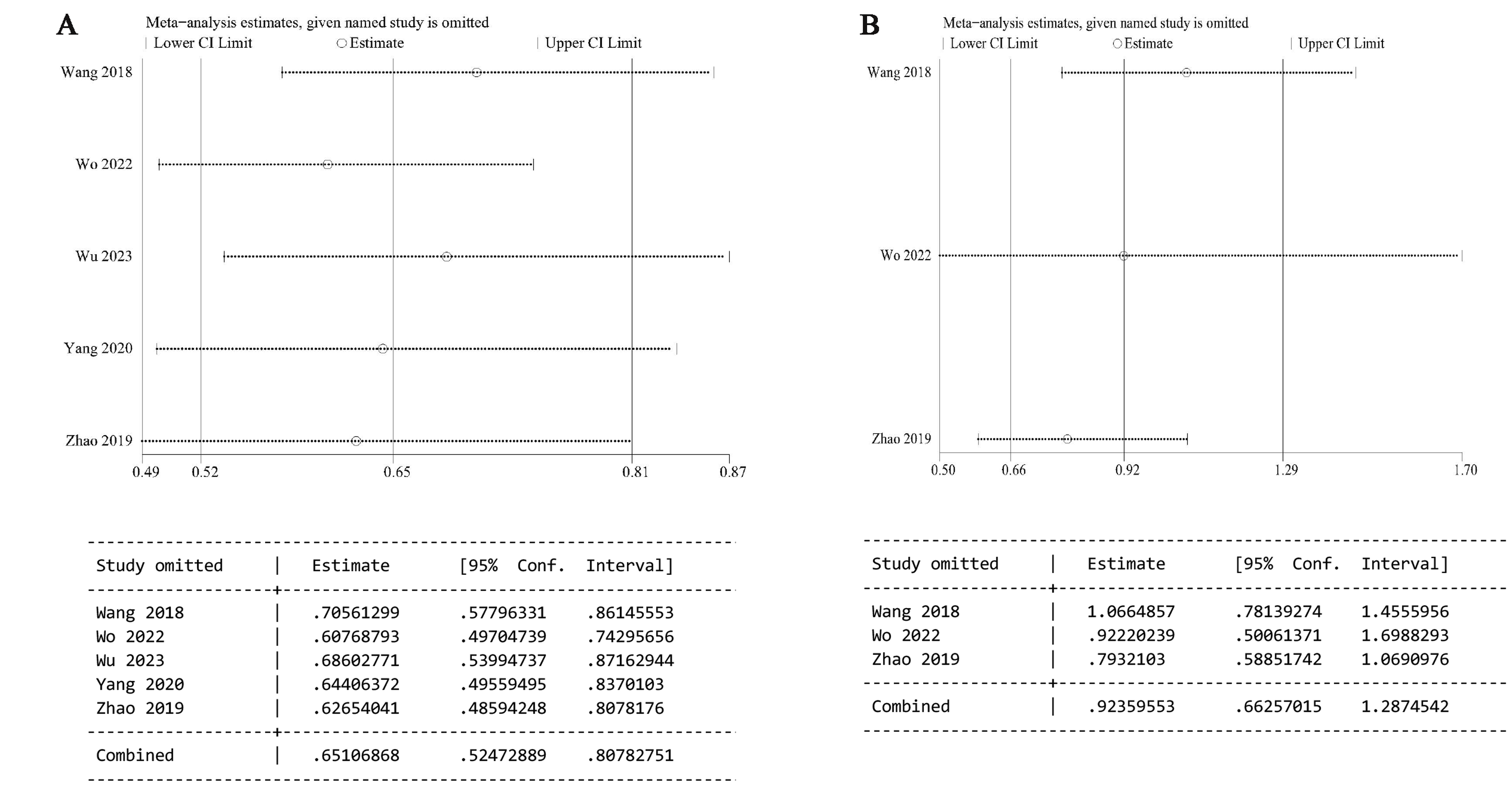

Supplement: Supplementary Figure 6 — Sensitivity analysis of OS and DFS. [file Image6.tif]

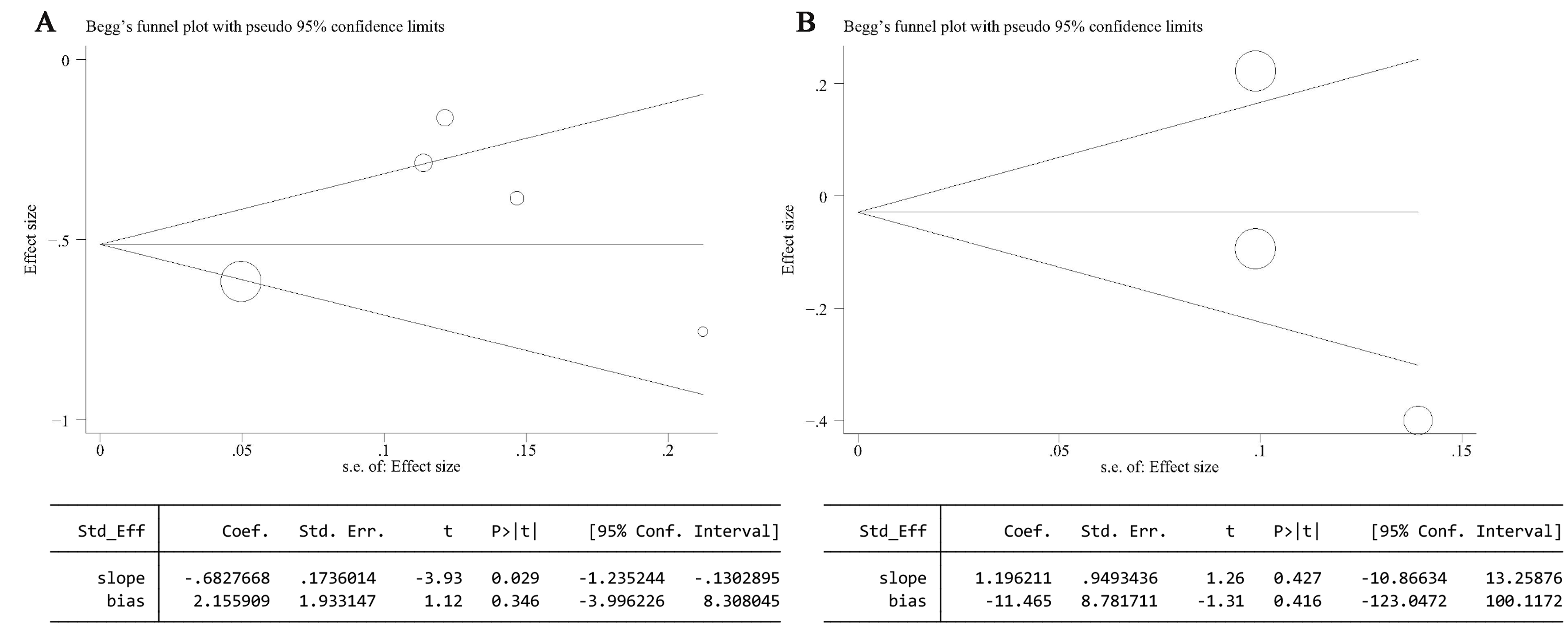

Supplement: Supplementary Figure 7 — Publication bias of OS and DFS. [file Image7.tif]
