# Supplementary material for: Dissection of 4L lymph node for left-sided non-small cell lung cancer: a meta-analysis
Source: Front Oncol. 2025 Jun 9;15:1583508. doi: 10.3389/fonc.2025.1583508 (PMC12183196; doi:10.3389/fonc.2025.1583508)
Supplement: Supplementary Table 2 — Search strategy. [file Table2.docx]

**Table S2** Search strategy

| **PubMed**  The database was searched on April 15, 2025, n=103.  Search Strategy:  (4L[Title/Abstract] OR left lower paratracheal[Title/Abstract]) AND (Left[Title/Abstract] OR Left side[Title/Abstract] OR Left-sided[Title/Abstract]) AND (Lung neoplasms[Title/Abstract] OR Pulmonary Neoplasms[Title/Abstract] OR Neoplasms, Lung[Title/Abstract] OR Lung Neoplasm[Title/Abstract] OR Neoplasm, Lung[Title/Abstract] OR Neoplasms, Pulmonary[Title/Abstract] OR Neoplasm, Pulmonary[Title/Abstract] OR Pulmonary Neoplasm[Title/Abstract] OR Lung Cancer[Title/Abstract] OR Cancer, Lung[Title/Abstract] OR Cancers, Lung[Title/Abstract] OR Lung Cancers[Title/Abstract] OR Pulmonary Cancer[Title/Abstract] OR Cancer, Pulmonary[Title/Abstract] OR Cancers, Pulmonary[Title/Abstract] OR Pulmonary Cancers[Title/Abstract] OR Cancer of the Lung[Title/Abstract] OR Cancer of Lung[Title/Abstract]) |
| --- |
| **Web of Science**  The database was searched on April 15, 2025, n=57.  Search Strategy:  1 TOPIC: (“4L”) (2438)  2 TOPIC: (“Left” OR “Left side” OR “Left-sided”) (1343157)  3 TOPIC: (“Lung neoplasms” OR “Pulmonary Neoplasms” OR “Neoplasms, Lung” OR “Lung Neoplasm” OR “Neoplasm, Lung” OR “Neoplasms, Pulmonary” OR “Neoplasm, Pulmonary” OR “Pulmonary Neoplasm” OR “Lung Cancer” OR “Cancer, Lung” OR “Cancers, Lung” OR “Lung Cancers” OR “Pulmonary Cancer” OR “Cancer, Pulmonary” OR “Cancers, Pulmonary” OR “Pulmonary Cancers” OR “Cancer of the Lung” OR “Cancer of Lung”) (414018)  4 #1 AND #2 AND #3 (42) |
| **EMBASE**  The database was searched on April 15, 2025, n=174.  Search Strategy:  ('4L':ti,ab,kw OR 'left lower paratracheal':ti,ab,kw) AND ('Left':ti,ab,kw OR 'Left side':ti,ab,kw OR 'Left-sided':ti,ab,kw) AND ('Lung neoplasms':ti,ab,kw OR 'Pulmonary Neoplasms':ti,ab,kw OR 'Neoplasms, Lung':ti,ab,kw OR 'Lung Neoplasm':ti,ab,kw OR 'Neoplasm, Lung':ti,ab,kw OR 'Neoplasms, Pulmonary':ti,ab,kw OR 'Neoplasm, Pulmonary':ti,ab,kw OR 'Pulmonary Neoplasm':ti,ab,kw OR 'Lung Cancer':ti,ab,kw OR 'Cancer, Lung':ti,ab,kw OR 'Cancers, Lung':ti,ab,kw OR 'Lung Cancers':ti,ab,kw OR 'Pulmonary Cancer':ti,ab,kw OR 'Cancer, Pulmonary':ti,ab,kw OR 'Cancers, Pulmonary':ti,ab,kw OR 'Pulmonary Cancers':ti,ab,kw OR 'Cancer of the Lung':ti,ab,kw OR 'Cancer of Lung':ti,ab,kw) |
| **Cochrane Library**  The database was searched on April 15, 2025, n=51.  Search Strategy:  (“4L” OR “left lower paratracheal”): ti,ab,kw AND (“Left” OR “Left side” OR “Left-sided”): ti,ab,kw AND (“Lung neoplasms” OR “Pulmonary Neoplasms” OR “Neoplasms, Lung” OR “Lung Neoplasm” OR “Neoplasm, Lung” OR “Neoplasms, Pulmonary” OR “Neoplasm, Pulmonary” OR “Pulmonary Neoplasm” OR “Lung Cancer” OR “Cancer, Lung” OR “Cancers, Lung” OR “Lung Cancers” OR “Pulmonary Cancer” OR “Cancer, Pulmonary” OR “Cancers, Pulmonary” OR “Pulmonary Cancers” OR “Cancer of the Lung” OR “Cancer of Lung”): ti,ab,kw - (Word variations have been searched) |
| **Scopus**  The database was searched on April 15, 2025, n=46.  Search Strategy:  TITLE-ABS-KEY ((“4L”) and (“Left” OR “Left side” OR “Left-sided”) and (“Lung neoplasms” OR “Pulmonary Neoplasms” OR “Neoplasms, Lung” OR “Lung Neoplasm” OR “Neoplasm, Lung” OR “Neoplasms, Pulmonary” OR “Neoplasm, Pulmonary” OR “Pulmonary Neoplasm” OR “Lung Cancer” OR “Cancer, Lung” OR “Cancers, Lung” OR “Lung Cancers” OR “Pulmonary Cancer” OR “Cancer, Pulmonary” OR “Cancers, Pulmonary” OR “Pulmonary Cancers” OR “Cancer of the Lung” OR “Cancer of Lung”)) |

**Note:** The combined text and medical subject heading (MeSH) terms used were: “4L”, “Left” and “Lung cancer”.
