# Supplementary material for: Dissection of 4L lymph node for left-sided non-small cell lung cancer: a meta-analysis
Source: Front Oncol. 2025 Jun 9;15:1583508. doi: 10.3389/fonc.2025.1583508 (PMC12183196; doi:10.3389/fonc.2025.1583508)
Supplement: Supplementary Table 3 — Methodological quality assessments of the included studies. [file Table3.docx]

Table S3 Methodological quality assessments of the included studies.

| **Study** | | **Selection** | | | | **Comparability ^d^** | **Outcome** | | | **Total score** |
| --- | --- | --- | --- | --- | --- | --- | --- | --- | --- | --- |
|  |  | **Exposed cohort ^a^** | **Nonexposed cohort ^b^** | **Ascertainment of exposure** | **Outcome of interest ^c^** |  | **Assessment of outcome** | **Length of follow-up ^e^** | **Adequacy of follow-up** |  |
| 2018 | Wang[7] | * | * | * | * | ** | * | * | * | 9 |
| 2019 | Zhao[13] | * | * | * | * | ** | * |  | * | 8 |
| 2020 | Yang[14] | * | * | * | * | ** | * | * | * | 9 |
| 2021 | Gryszko[15] | * | * | * | * | ** | * | * | * | 9 |
| 2022 | Wo[16] | * | * | * | * | ** | * | * | * | 9 |
| 2023 | Wu[] | * | * | * | * | ** | * | * | * | 9 |

Note: ^a^ Representativeness of the exposed cohort;

^b^ Selection of the non-exposed cohort;

^c^ Demonstration that outcome of interest was not present at start of study;

^d^ Comparability of cohorts on the basis of the design or analysis;

^e^ Was follow-up long enough for outcomes to occur.
