# Supplementary material for: Dissection of 4L lymph node for left-sided non-small cell lung cancer: a meta-analysis
Source: Front Oncol. 2025 Jun 9;15:1583508. doi: 10.3389/fonc.2025.1583508 (PMC12183196; doi:10.3389/fonc.2025.1583508)
Supplement: Supplementary Table 4 — GRADE Quality assessment by therapeutic strategy and study design for the outcomes of survival and postoperative complications. [file Table4.docx]

**Table S4** GRADE Quality assessment by therapeutic strategy and study design for the outcomes of survival and postoperative complications.

| **Primary  outcomes** | **No. of Studies** | **No. of participants** | | **Differences ^a^（95%CI）** |  | **Quality assessment** | | | | **Quality** |
| --- | --- | --- | --- | --- | --- | --- | --- | --- | --- | --- |
|  |  | **4L LND+** | **4L LND-** |  | **Risk of**  **Bias ^b^** | **Inconsistency** | **Indirectness** | **Imprecision** | **Publication bias ^c^** |  |
| **Survival** |  |  |  |  |  |  |  |  |  |  |
| OS | 5 | 1986 | 2267 | 0.63 [0.58, 0.68] | Low | Serious (-1) | No indirectness | No imprecision | Unlikely | Very low |
| OSR |  |  |  |  |  |  |  |  |  |  |
| 1-year | 4 | 1242/1327 | 1455/1608 | 0.97 [0.95, 0.99] | Low | No inconsistency | No indirectness | No imprecision | Unlikely | Low |
| 2-year | 4 | 1125/1327 | 1261/1608 | 0.93 [0.90, 0.97] | Low | No inconsistency | No indirectness | No imprecision | Unlikely | Low |
| 3-year | 4 | 1057/1327 | 1131/1608 | 0.90 [0.86, 0.93] | Low | Serious (-1) | No indirectness | No imprecision | Unlikely | Very low |
| 4-year | 4 | 989/1327 | 1020/1608 | 0.87 [0.83, 0.92] | Low | No inconsistency | No indirectness | No imprecision | Unlikely | Low |
| 5-year | 4 | 892/1327 | 941/1608 | 0.90 [0.85, 0.95] | Low | Serious (-1) | No indirectness | No imprecision | Unlikely | Very low |
| DFS | 2 | 550/1327 | 831/1608 | 0.82 [0.70, 0.96] | Low | Serious (-1) | No indirectness | No imprecision | Unlikely | Very low |
| DFSR |  |  |  |  |  |  |  |  |  |  |
| 1-year | 3 | 831/1010 | 1040/1291 | 0.94 [0.90, 0.98] | Low | No inconsistency | No indirectness | No imprecision | Unlikely | Low |
| 2-year | 3 | 694/1010 | 842/1291 | 0.93 [0.83, 1.03] | Low | Serious (-1) | No indirectness | No imprecision | Unlikely | Very low |
| 3-year | 3 | 621/1010 | 734/1291 | 0.91 [0.79, 1.04] | Low | Serious (-1) | No indirectness | No imprecision | Unlikely | Very low |
| 4-year | 3 | 575/1010 | 6791291 | 0.92 [0.77, 1.10] | Low | Serious (-1) | No indirectness | No imprecision | Unlikely | Very low |
| 5-year | 3 | 525/1010 | 628/1291 | 0.91 [0.71, 1.17] | Low | Serious (-1) | No indirectness | No imprecision | Unlikely | Very low |
| **Postoperative complications and recurrence** |  |  |  |  |  |  |  |  |  |  |
| Chylothorax | 3 | 13/995 | 11/1069 | 1.28 [0.58, 2.84] | Low | No inconsistency | No indirectness | Serious (-1) | Unlikely | Very low |
| Pneumonia | 3 | 35/995 | 28/1069 | 1.29 [0.79, 2.10] | Low | No inconsistency | No indirectness | No imprecision | Unlikely | Low |
| Hemorrhage | 2 | 8/535 | 2/609 | 4.67 [0.93, 23.46] | Low | No inconsistency | No indirectness | Serious (-1) | Unlikely | Very low |
| Air leak>7 days | 1 | 10/416 | 7/416 | 1.43 [0.55, 3.72] | Low | No inconsistency | No indirectness | No imprecision | Unlikely | Low |
| Chest tube drain> 7 days | 2 | 65/876 | 49/876 | 1.33 [0.93, 1.88] | Low | No inconsistency | No indirectness | Serious (-1) | Unlikely | Very low |
| Heart failure | 1 | 2/416 | 2/416 | 1.00 [0.14, 7.07] | Low | No inconsistency | No indirectness | Serious (-1) | Unlikely | Very low |
| Recurrent nerve injury | 1 | 3/416 | 1/416 | 3.00 [0.31, 28.72] | Low | No inconsistency | No indirectness | Serious (-1) | Unlikely | Very low |
| Hoarseness | 1 | 5/416 | 4/416 | 1.25 [0.34, 4.63] | Low | No inconsistency | No indirectness | Serious (-1) | Unlikely | Very low |
| Bronchopleural fistula | 2 | 5/579 | 5/653 | 1.10 [0.32, 3.76] | Low | No inconsistency | No indirectness | Serious (-1) | Unlikely | Very low |
| Deep venous thrombosis | 1 | 1/416 | 3/416 | 0.33 [0.03, 3.19] | Low | No inconsistency | No indirectness | Serious (-1) | Unlikely | Very low |
| Pulmonary embolism | 1 | 1/416 | 1/416 | 1.00 [0.06, 15.94] | Low | No inconsistency | No indirectness | Serious (-1) | Unlikely | Very low |
| Pneumothorax | 1 | 7/119 | 8/193 | 1.42 [0.53, 3.81] | Low | No inconsistency | No indirectness | Serious (-1) | Unlikely | Very low |
| Arrhythmia | 1 | 2/119 | 3/193 | 1.08 [0.18, 6.38] | Low | No inconsistency | No indirectness | Serious (-1) | Unlikely | Very low |
| Respiratory failure | 1 | 2/119 | 2/193 | 1.62 [0.23, 11.36] | Low | No inconsistency | No indirectness | Serious (-1) | Unlikely | Very low |
| Incision infection | 1 | 1/119 | 0/193 | 4.85 [0.20, 118.09] | Low | No inconsistency | No indirectness | Serious (-1) | Unlikely | Very low |
| Hydrothorax | 1 | 1/119 | 4/193 | 0.41 [0.05, 3.58] | Low | No inconsistency | No indirectness | Serious (-1) | Unlikely | Very low |
| Others | 1 | 4/119 | 2/193 | 3.24 [0.60, 17.44] | Low | No inconsistency | No indirectness | Serious (-1) | Unlikely | Very low |
| Overall recurrence | 2 | 207/831 | 314/831 | 0.49 [0.11, 2.24] | Low | Serious (-2) | No indirectness | No imprecision | Unlikely | Very low |
| Regional LN recurrence | 1 | 30/416 | 41/416 | 0.73 [0.47, 1.15] | Low | No inconsistency | No indirectness | No imprecision | Unlikely | Low |
| Supraclavicular or cervical LN recurrence | 1 | 11/416 | 14/416 | 0.79 [0.36, 1.71] | Low | No inconsistency | No indirectness | Serious (-1) | Unlikely | Very low |

**Abbreviations:** OS: overall survival; OSR: overall survival rate; DFS: disease-free survival; DFSR: disease-free survival rate; 4L: Left Lower Paratracheal; LN: lymph node; LND: Lymph node dissection; CI: confidence interval.

^a^ Differences: hazard ratio (HR) for OS and DFS; risk ratios (RR) for OSR, DFSR, postoperative complications, overall recurrence, regional LN recurrence and supraclavicular or cervical LN recurrence.

^b^ Risk of bias assessed using the Newcastle-Ottawa Scale (NOS) for non-randomized studies and Jadad scale for randomized controlled trials.

^c^ Publication bias was assessed by Egger’s and Begg’s tests.
